# FOXP2 drives neuronal differentiation by interacting with retinoic acid signaling pathways

**DOI:** 10.3389/fncel.2014.00305

**Published:** 2014-09-26

**Authors:** Paolo Devanna, Jeroen Middelbeek, Sonja C. Vernes

**Affiliations:** ^1^Language and Genetics Department, Max Planck Institute for PsycholinguisticsNijmegen, Netherlands; ^2^Laboratory of Pediatric Oncology, Radboud Institute for Molecular Life Sciences, Radboud UniversityNijmegen, Netherlands; ^3^Donders Institute for Brain, Cognition and Behaviour, Radboud UniversityNijmegen, Netherlands

**Keywords:** language, neurite outgrowth, forkhead transcription factors, FOXP2, retinoic acid, neuron differentiation, SH-SY5Y cells, neuronal migration

## Abstract

*FOXP2* was the first gene shown to cause a Mendelian form of speech and language disorder. Although developmentally expressed in many organs, loss of a single copy of *FOXP2* leads to a phenotype that is largely restricted to orofacial impairment during articulation and linguistic processing deficits. Why perturbed FOXP2 function affects specific aspects of the developing brain remains elusive. We investigated the role of FOXP2 in neuronal differentiation and found that *FOXP2* drives molecular changes consistent with neuronal differentiation in a human model system. We identified a network of *FOXP2* regulated genes related to retinoic acid signaling and neuronal differentiation. *FOXP2* also produced phenotypic changes associated with neuronal differentiation including increased neurite outgrowth and reduced migration. Crucially, cells expressing FOXP2 displayed increased sensitivity to retinoic acid exposure. This suggests a mechanism by which FOXP2 may be able to increase the cellular differentiation response to environmental retinoic acid cues for specific subsets of neurons in the brain. These data demonstrate that *FOXP2* promotes neuronal differentiation by interacting with the retinoic acid signaling pathway and regulates key processes required for normal circuit formation such as neuronal migration and neurite outgrowth. In this way, FOXP2, which is found only in specific subpopulations of neurons in the brain, may drive precise neuronal differentiation patterns and/or control localization and connectivity of these FOXP2 positive cells.

## Introduction

Mutations in the *FOXP2* gene are known to cause rare forms of speech and language disorder, the first report of which was the KE family in 2001 (Lai et al., 2001). Additional *FOXP2* gene disruptions have since been identified in a number of unrelated individuals or families with similar phenotypes (Macdermot et al., 2005; Shriberg et al., 2006; Palka et al., 2011; Rice et al., 2012; Žilina et al., 2012). Affected individuals all carry heterozygous mutations in *FOXP2*, meaning that they still have one functional copy of the gene. A complete loss of *FOXP2* is thought to be lethal for humans, as it is in mouse models, likely due to developmental defects in multiple organs (Lu et al., 2002; Shu et al., 2007; Rousso et al., 2012). Although developmentally expressed in many tissues including the brain, lung, and heart, reduced levels of functional *FOXP2* results in a phenotype that is largely restricted to orofacial impairment during articulation and linguistic processing deficits in patients (Vargha-Khadem et al., 1995; Alcock et al., 2000; Watkins et al., 2002a). This highly specific phenotype suggests that particular aspects of the nervous system have a lower tolerance for *FOXP2* reduction than other tissues, such as the heart or lung. The effect of *FOXP2* mutation on brain structure and function has been studied in members of the KE family and no gross abnormalities have been found (Watkins et al., 1999; Lai et al., 2003). Instead only subtle effects are observed including changes to gray matter density in regions of the cortex, thalamus and striatum and functional activation differences during language tasks in a language related area of the cortex (Broca's area) and the striatum (Watkins et al., 1999, 2002b; Liegeois et al., 2003). Hence, the activity of FOXP2 in a subset of neurons throughout the brain is thought to be essential for the proper development of neural networks important for normal speech and language.

*FOXP2* encodes a Forkhead-box (FOX) transcription factor (Vernes et al., 2006). Related FOX transcription factors, such as FOXP1, have also been implicated in cognitive disorders. FOXP1 and FOXP2 have high sequence homology, display overlapping expression patterns and can form functional heterodimers to bind target DNA. However, mutations in FOXP1 result in a broad spectrum of cognitive impairments in patients including severe intellectual disability, gross motor delay and autism spectrum disorder (Hamdan et al., 2010; Horn et al., 2010; Horn, 2011; O'roak et al., 2011; Palumbo et al., 2013). Linguistic processing defects have not been found in FOXP1 patients and, although speech delay is sometimes seen, this is thought to be more related to general cognitive impairments and motor problems, rather than a speech/language specific effect (Bacon and Rappold, 2012). This highlights the importance of directly studying FOXP2 function at a molecular level, since it seems to be playing a unique and critical role in language related circuitry that is different to even its most closely related family member, FOXP1.

FOXP2 is expressed in a number of brain structures including the cortex, basal ganglia, thalamus, cerebellum, midbrain, and medulla (Ferland et al., 2003; Lai et al., 2003). Previous studies have identified a range of genomic regions bound by FOXP2 in human brain tissue, human neuron-like cells and embryonic mouse brain (Spiteri et al., 2007; Vernes et al., 2007, 2008, 2011). The putative FOXP2 targets identified in these studies were known to act in pathways ranging from GABA signaling, Wnt pathway signaling, neurogenesis, neuronal differentiation and cell migration (Spiteri et al., 2007; Vernes et al., 2007, 2011). Consistent with the high number of FOXP2 target genes involved in neurite outgrowth, Foxp2 (lowercase denotes the mouse homolog) was found to promote the growth and branching of neurites in medium spiny neurons (MSN) of the developing mouse striatum (Vernes et al., 2011) and affect spine density in mouse cortical neurons (Sia et al., 2013). Moreover, *Foxp2* gene disruption leads to abnormal neuronal activity and altered striatal plasticity in mice (Groszer et al., 2008; French et al., 2012), implicating this protein in controlling not only the morphology but also the connectivity of neurons. Recently, an early developmental role for Foxp2 has been proposed in which Foxp2 enhances the transition from radial glial precursor to cortical neuron in the mouse brain (Tsui et al., 2013).

Given the developmental importance of FOXP2, and its putative roles in pathways such as neurogenesis, neuronal migration and neurite outgrowth, we investigated if and how FOXP2 contributes to human neuronal differentiation. For this purpose, we chose a well-defined model of human neuronal differentiation, the SHSY5Y human neuron-like cell line. SHSY5Y cells can switch from a proliferative to a more differentiated neuronal phenotype by stimulation with differentiation inducing compounds, such as all-trans retinoic acid (RA) and/or growth factors such as brain derived neurotrophic factor (BDNF) (Voigt and Zintl, 2003; Agholme et al., 2010; Lopes et al., 2010; Dwane et al., 2013). In this model, these factors reduce cell migration, increase expression of pro-neural gene markers, reduce expression of pluripotency markers and promote the growth and extension of long and extensively branched neurites, indicative of neuronal differentiation (Voigt and Zintl, 2003; Lopes et al., 2010).

This study demonstrates that FOXP2 induces molecular and phenotypic features of neuronal differentiation. We show that FOXP2 mediates changes in gene expression resembling those occurring during differentiation to drive a more neuronal phenotype. FOXP2 controls similar gene expression programs to those that are regulated by RA, and increases the expression of a retinoic acid receptor central to RA signaling (RARβ). Interestingly, our results suggest that FOXP2 increases the sensitivity of cells to RA, influencing their molecular properties to drive neuronal differentiation. The interaction of FOXP2 with retinoic acid pathways, which are key to normal brain development and patterning, may represent an important mechanism in controlling cell type specific differentiation patterns and connectivity in language related circuitry in the developing brain.

## Materials and methods

### Cell culture and reagents

Stable SHSY5Y cells expressing human FOXP2 or the empty vector (EMPTY) generated previously (Vernes et al., 2007) were grown at 37°C in the presence of 5% CO_2_ in growth media; DMEM:F12 media (Invitrogen, Carlsbad, CA, USA) supplemented with 10% Fetal Calf Serum (FCS; Sigma, St Louis, MO, USA), 2 mM L-glutamine (Sigma, St Louis, MO, USA), 1% Non-essential amino acids (NEAA; Invitrogen, Carlsbad, CA, USA) and 2 mM Penicillin/Streptomycin (Sigma, St Louis, MO, USA). Neuronal differentiation protocol involved treating cells with 10 μM all-trans retinoic acid (RA) in low serum media (DMEM:F12, 2% Fetal Calf Serum, 2 mM L-glutamine, 2 mM Penicillin/Streptomycin, 1% NEAA) for 5 days, followed by a further 10 days of treatment with 50 ng/mL BDNF in serum free media. All other retinoic acid treatments were performed in low serum media. Transfections were carried out with Transfast® (Promega, Madison, WI, USA), according to the manufacturers' instructions.

### Quantitative RT-PCR

Total RNA was extracted from cells harvested in TRIzol® reagent using the RNeasy kit (QIAGEN, Venlo, NL) according to manufacturers instructions. RNA was extracted from three biological replicates of SHSY5Y cells stably transfected either with human *FOXP2* or the empty control vector following culture in growth media (Day 0) or following the differentiation protocol (Day 5, 10 or 15). Reverse transcription was performed as described previously (Vernes et al., 2007).

PCR reactions utilized SYBR Green supermix (BioRad, Hercules, CA, USA) as described previously (Vernes et al., 2007). Primers specific for candidate genes and the control housekeeping genes *CYPA* and *POLR2F* are described in Supplementary Table S1. Quantitative PCR reactions were performed on the CFX96 Real-Time PCR Detection System (BioRad, Hercules, CA, USA) according to manufacturers' instructions.

Melting curve analysis was performed to assess the specificity of the amplification. Data analysis was performed using CFX manager software (BioRad, Hercules, CA, USA), and quantification was performed via the comparative CT method (Livak and Schmittgen, 2001). Fold changes are reported following normalization to the geometric mean of two internal controls; *CYPA* and *POLR2F* (Hoerndli et al., 2004; Agholme et al., 2010). Data are expressed as mean ± standard deviation. Statistical significance was assessed using students *t*-tests (two-tailed) for pairs of means or ANOVA test for groups of three or more means followed by *post-hoc* Bonferroni, Tukey or Dunnett calculation as specified.

### Retinoic acid dose response

SHSY5Y control (EMPTY) and FOXP2 cells were grown in low serum media (as detailed above) and were treated with increasing doses of RA (from 0.001 to 10 μM) or no added RA for 3 days. Total RNA was extracted from three biological replicates per treatment and reverse transcribed as described above. RT-PCR reactions were performed as described above. All fold changes are reported relative to expression levels in the SHSY5Y control (EMPTY) cell line in low serum media, following internal normalization to *POLR2F*. Statistical significance was assessed using ANOVA test followed by *post-hoc* Bonferroni calculation.

### Neurite outgrowth analysis

Cells were plated onto poly-L-lysine coated coverslips at 3.3 × 10^4^ cells per well. Cells were fixed using 4% Paraformaldehyde solution for 15 min at room temperature and permeabilised in wash solution (0.1% Triton X-100 in TBS). Antibodies were diluted in Blocking Solution (1% Fish Gelatine, 0.1% Triton X-100, 5% BSA in PBS). Cells were co-stained at 4°C overnight, using an anti-MAP2 rabbit polyclonal antibody (Chemicon, Temecula, CA, USA). Cells were incubated with anti-rabbit FITC (Alexa Fluor 488, Molecular Probes, Carlsbad, CA, USA) for 1 h, shaking under limited light exposure. Nuclei were visualized using mounting media containing a DAPI counterstain (VectaShield). Cells were viewed on a Zeiss Axiovert A-1 fluorescence inverted microscope. Images were captured using a Zeiss AxioCam MRm camera and Zen Software (Zeiss, Oberkochen, GER), and analyzed using the neurite outgrowth function of Metamorph Version 7.8 (Molecular Devices, Sunnyvale, CA). Statistical significance was assessed using students *t*-tests (two-tailed). Data are expressed as the mean ± standard error of the mean (s.e.m.).

### Cell migration analysis

Gap closure assays were performed according to manufacturer's recommendations (Ibidi, Martinsried, GER). 2 × 10^4^ control (EMPTY) or FOXP2 expressing cells (FOXP2) were seeded per insert well (Ibidi Culture Inserts #80209) and cultured overnight at 37°C in the presence of 5% CO_2_. Culture media was supplemented with 1 μM Aphidicolin to inhibit cell division that would obscure the effects of FOXP2 expression on migration. Cells were allowed to migrate after removal of the inserts, and gap closure was imaged for 72 h by time lapse microscopy (with one photo taken every 10 min).

Gap closure speed was determined by automated measurements of the gap-size throughout the series of images, using an ImageJ image analysis routine (developed by K. Jalink, NKI, Amsterdam, The Netherlands). In short, images were normalized with respect to intensity/contrast and subjected to the variance filter in order to distinguish moving cells from the remaining gap. The area of the remaing gap was quantified in each successive image and plotted against time. Quantification was performed by taking the migration values after 25, 50, and 75% gap closure for non-FOXP2 expressing cells and comparing the migration values for FOXP2 positive cells at the same time points. Data are expressed as mean of 6 image sets ± standard deviation. Statistical significance was assessed using Student's *t*-tests (two-tailed).

### Gene ontology analysis

Gene Ontology (GO) analysis was performed using the Webgestalt program (http://bioinfo.vanderbilt.edu/webgestalt/). Over representation of gene ontology categories was determined via hypergeometric testing using Benjamini and Hochberg multiple testing correction (Zhang et al., 2005).

## Results

### FOXP2 induces expression of neuronal differentiation markers

**Figure 1:**
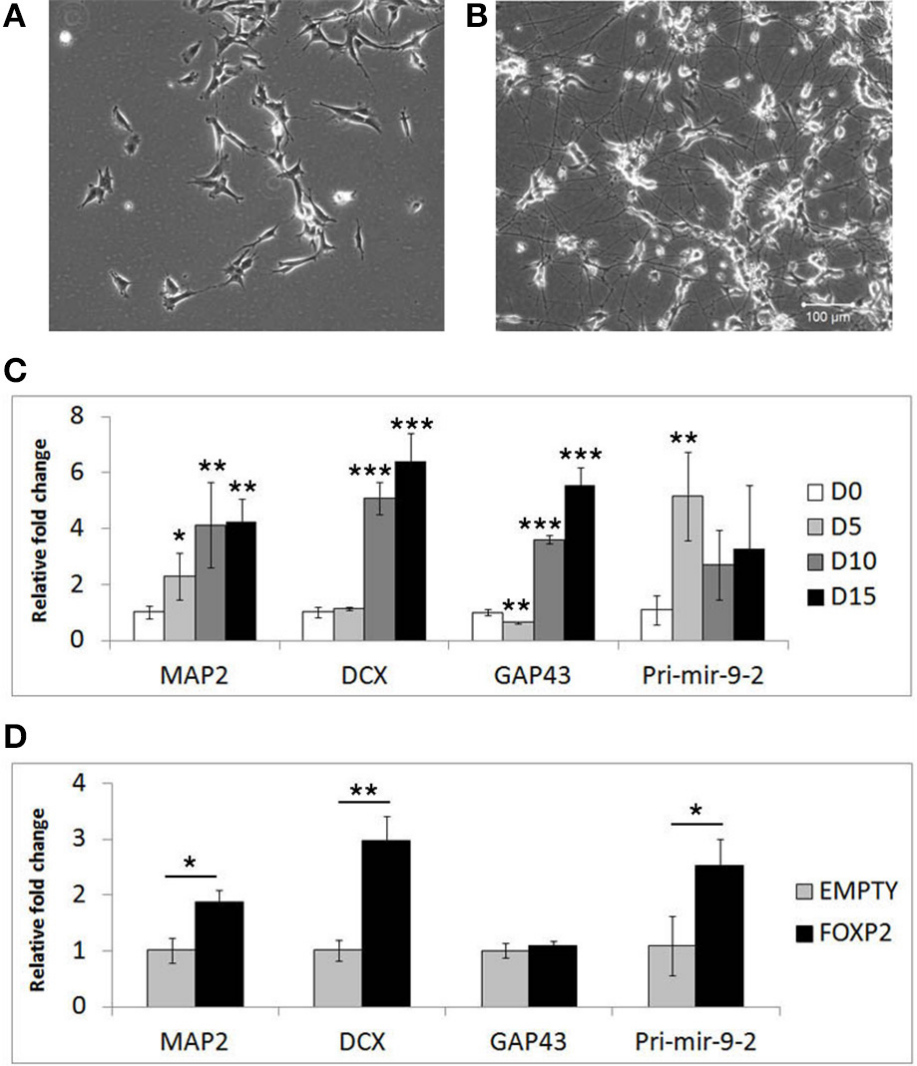
**Markers of neuronal differentiation show increased expression in the presence of FOXP2**. SHSY5Y control cells (EMPTY) were differentiated using the RA/BDNF protocol outlined in Materials and Methods for 15 days. **(A)** Cell morphology at Day 0, i.e., undifferentiated SHSY5Y cells in normal growth media. **(B)** After 15 days exposure to sequential RA and BDNF treatment to induce neuronal-like differentiation, dramatic morphological changes can be seen including smaller cell bodies and substantial increases in neurite outgrowth and complexity. Scale bar = 100 μm. **(C)** Gene expression changes in SHSHY5Y control (EMPTY) cells were assessed before (Day 0; D0) and during differentiation (Day 5, Day 10, and Day 15). The differentiation markers *MAP2, DCX, GAP43*, and *mir-9-2* were significantly upregulated after 5–10 days treatment. Significant differences between groups was calculated using an ANOVA test followed by *post-hoc* Dunnett calculation. ^*^p < 0.05, ^**^p < 0.01, ^***^p < 0.001. **(D)** Gene expression changes were assessed in SHSY5Y cells stably transfected with either an empty vector (EMPTY) or human FOXP2 (FOXP2) without differentiation (i.e., Day 0). FOXP2 expression resulted in upregulation of the differentiation markers *MAP2, DCX* and *mir-9-2* (primary transcript) compared to empty vector transfected cells (EMPTY) when cells were maintained in normal growth media (without added RA or BDNF). Statistical significance was assessed using student's *t*-tests (two-tailed). ^*^p < 0.05, ^**^p < 0.01. All data are the average of three biological replicates expressed as mean ± standard deviation.

Given that FOXP2 has previously been implicated in neurogenesis and neuronal development, we wanted to determine if FOXP2 affects the differentiation of neuron-like cells. SHSY5Y human neuron-like cells were derived from a neuroblastoma biopsy and are widely used for studying neuronal differentiation (Biedler et al., 1973; Messi et al., 2008; Lopes et al., 2010; Xie et al., 2010; Dwane et al., 2013). We established a neuronal differentiation protocol in which cells are treated for 5 days with RA in low serum (2% FCS) media, followed by a further 10 days of treatment with BDNF in the absence of RA or serum. This protocol led to dramatic morphological differentiation (Figures 1A,B) and strong and reproducible increases in expression levels of neuronal differentiation markers including microtubule associated protein (*MAP2*), doublecortin (*DCX*) growth associated protein 43 (*GAP43*) and the neuronal microRNA mir-9 (Figure 1C).

We next set out to test whether FOXP2 could drive similar gene expression changes when the cells were maintained in normal growth media (i.e., without the addition of RA or BDNF supplements). Given that SHSY5Y cells do not express FOXP2 endogenously, we used SHSY5Y cells that were made to stably express human FOXP2 (FOXP2) or an empty vector control (EMPTY) (Vernes et al., 2007) to determine the effect of FOXP2 in this system. We found that expression of FOXP2 protein alone was sufficient to induce significant increases in neuronal markers of differentiation, including *MAP2, DCX*, and the pro-neural microRNA mir-9-2 (Figure 1D).

### FOXP2 mediated gene expression changes are consistent with those occurring during neuronal differentiation

In addition to well-known differentiation markers such as *MAP2* and *DCX*, neuronal differentiation involves widespread changes in gene expression programs. We surveyed the literature for additional differentiation related genes. We assembled a list of 45 genes (Supplementary Table S1) including retinoic acid receptors (*RARs, RXRs*, and *ROR*s), genes known to respond to retinoic acid or BDNF stimulation (e.g., as *ASCL1, ID1-3, BCL-2*), genes involved in neurite outgrowth and migration (e.g., *NAV2, NEDD9*) as well as putative FOXP2 target genes including a retinoic acid receptor (*RAR*β), transcription factors (*BATF3, HOXD10*, and *ETV1*), and neuronal differentiation factors (*NEUROD2, NEUROD6* and *FGF1*) (Spiteri et al., 2007; Vernes et al., 2007, 2011).

**Figure 2:**
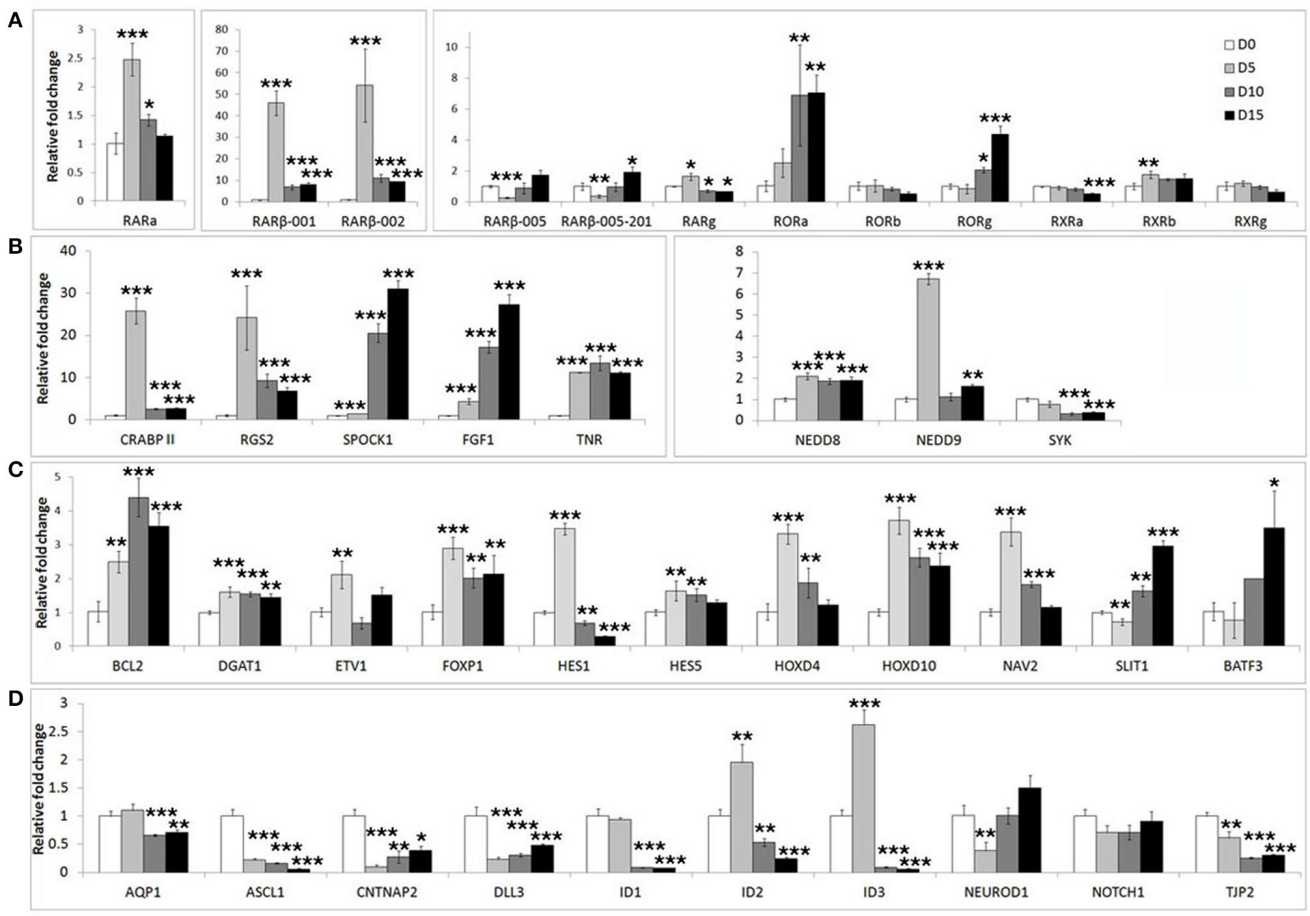
**Widespread gene expression changes are induced during neuron-like differentiation of human SHSY5Y cells**. SHSY5Y (EMPTY) control cells (which do not express any FOXP2 protein) were induced to differentiate via sequential treatment with RA and BDNF. Gene expression changes were assessed before (Day 0) and during differentiation (Day 5, Day 10, and Day 15). To observe the effect of differentiation, all gene expression levels were normalized to their own expression at Day 0. **(A,B)** Massive inductions were observed for a subset of genes including *RAR*β (~50 fold), *CRABPII, FGF1*, and *RGS2* (~25 fold). **(C)** Many other genes were more mildly (2–7 fold), but significantly induced. **(D)** A few genes were strongly downregulated during differentiation including *ASCL1, CNTNAP2*, and *DLL3* (80–90% reduction in expression). *NEUROD2, NEUROD6*, and *DLL1* could not be assessed as transcripts could not be detected in any samples, suggesting that these genes are not expressed in SHSY5Y cells. Data are the average of three biological replicates expressed as mean ± standard deviation. Significant differences between groups was calculated using an ANOVA test followed by *post-hoc* Dunnett calculation. ^*^p < 0.05, ^**^p < 0.01, ^***^p < 0.001.

We first assessed the expression of these genes during neuronal differentiation of control cells. Expression levels for these genes were determined in the SHSY5Y control cell line (EMPTY) during the differentiation protocol at the same four time points as before; Day 0, Day 5, Day 10, and Day 15. Significant changes were observed for the majority of genes tested (Figure 2). The most strongly affected genes included retinoic acid receptor β (*RAR*β; ~50-fold upregulation), cellular retinoic acid binding protein 2 (*CRABPII*; ~25-fold upregulation), regulator of G-protein signaling 2 (*RGS2*; ~23-fold upregulation), fibroblast growth factor 1 (*FGF1*; ~30-fold upregulation), achaete-scute complex homolog 1 (*ASCL1*; ~80% downregulation) and Delta-like ligand 3 (*DLL3*; ~80% downregulation). See Supplementary Table S2 for gene expression summary.

**Figure 3:**
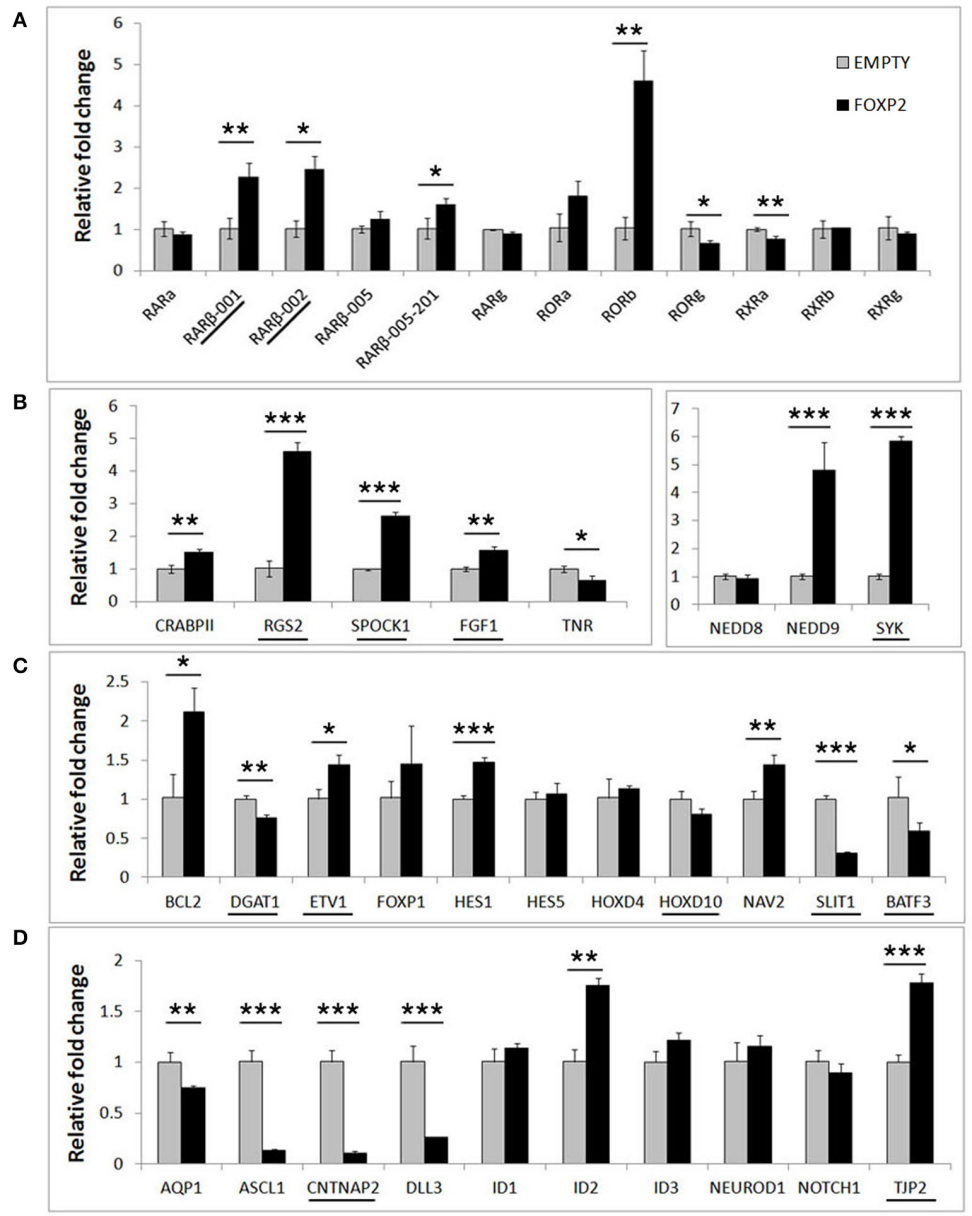
**FOXP2 regulates the expression of genes related to human neuronal differentiation**. The same genes tested in Figure 2 were assessed for regulation by FOXP2 in undifferentiated cells. Gene expression levels were compared between SHSY5Y control cells that do not express FOXP2 (EMPTY) or FOXP2 expressing counterparts (FOXP2), in normal growth media (i.e., equivalent to Day 0). **(A)** FOXP2 strongly upregulates multiple isoforms of the *RAR*β receptor and the orphan nuclear receptor (*RORb*). Mild but significant reductions in *RORg* and *RXRa* expression were also observed in FOXP2 expressing cells. **(B,C)** A number of other genes induced by differentiation were also upregulated by FOXP2 in the absence of differentiation stimulus, including *RGS2, NEDD9, SYK, BCL-2*, and *NAV2*. **(D)** In normal growth media, FOXP2 strongly downregulated *ASCL1, CNTNAP2*, and *DLL3*, all of which are significantly repressed during neuronal differentiation. Underlined genes have shown evidence that FOXP2 binds directly to their promoter regions in ChIP assays (Spiteri et al., 2007; Vernes et al., 2007, 2011). Data are the average of three biological replicates expressed as mean ± standard deviation. Statistical significance was assessed using student's *t*-tests (two-tailed). ^*^p < 0.05, ^**^p < 0.01, ^***^p < 0.001.

In order to determine if FOXP2 was able to regulate this set of differentiation related genes, we then compared their expression levels between untreated SHSY5Y control cells (EMPTY) and FOXP2 expressing cells (FOXP2) when cells were maintained in normal growth media (equivalent to Day 0). More than half of the genes assayed displayed altered expression levels in response to FOXP2 expression (Figure 3), suggesting that FOXP2 is regulating a wide range of differentiation related genes even in normal growth media (i.e., without the addition of RA/BDNF supplements). Moreover these expression changes were similar to those induced by the RA/BDNF differentiation protocol in control (EMPTY) cells. Targets previously identified via FOXP2-ChIP, including *CNTNAP2, SLIT1, BATF3*, and *DLL3*, were significantly downregulated in response to FOXP2 expression. The pro-neural transcription factor *ASCL1* was strongly repressed and *TNR, AQP1*, and the retinoic receptors *RXRa* and *RORg* were mildly downregulated (Figure 3 and Supplementary Table S2). The majority of these genes were also downregulated during at least one time point of differentiation in control (EMPTY) cells (Figure 2; Supplementary Table S2).

FOXP2 has been widely described as a transcriptional repressor, however a number of genes, including some previously identified as direct FOXP2 targets, were upregulated upon FOXP2 expression (Figure 3). Retinoic acid receptors such as *ROR*β and two isoforms of the putative FOXP2 target gene *RAR*β (isoforms *RAR*β-*001, RAR*β-*002*) were upregulated 2–5-fold. Of note, *RAR*β has multiple promoters producing at least four different protein coding transcripts, but FOXP2 was previously only shown to bind to the shared *RAR*β-*001/002* promoter (Vernes et al., 2007). Interestingly one of the earliest genes expressed during retinoic acid induced differentiation, *NEDD9*, was very highly upregulated by FOXP2 (~5 fold increase). FOXP2 target genes previously identified via ChIP-Seq including *RGS2, SPOCK1, SYK, TJP2, FGF1*, and *ETV1* were upregulated, as were novel targets *NAV2, HES1*, and *BCL-2* (Figure 3). This demonstrates that FOXP2 has the capacity to cause target gene expression to increase as well as to decrease, and thus should not be referred to only as a transcriptional repressor. A summary of all FOXP2 mediated gene expression changes as well as all changes that occur during differentiation in control cells can be found in Supplementary Table S2.

Overall, the presence of FOXP2 is sufficient to drive gene expression changes that resemble those occurring during SHSY5Y neuronal differentiation induced by RA and BDNF. This includes upregulation of *RAR*β, a nuclear receptor central to RA signaling that directly regulates the expression of downstream gene networks. FOXP2 also affected the expression of a range of genes known to be involved in RA signaling including pro-neural transcription factors (such as *ASCL1*) and proteins that directly affect differentiation phenotypes (*NAV2* and *NEDD9*). These data demonstrate that the FOXP2 transcription factor activates a complex gene expression program that drives neuronal differentiation.

### FOXP2 positive cells are more responsive to retinoic acid

The effects of RA in the developing brain are highly dependent on concentration gradients and the level of RA that cells are exposed to can influence their differentiation response (Maden, 2002; White et al., 2007; Rhinn and Dolle, 2012). Moreover, during development cells display a “sensitive period” in which the effects of RA are most severe (Sive et al., 1990; Holson et al., 2001; Yamamoto et al., 2003; Luo et al., 2004). This suggests that the amount of available RA, as well as the internal molecular state of the cell, is important to regulate the response induced by RA. Thus, we investigated if the presence of FOXP2 affected the way cells responded to RA treatment.

**Figure 4:**
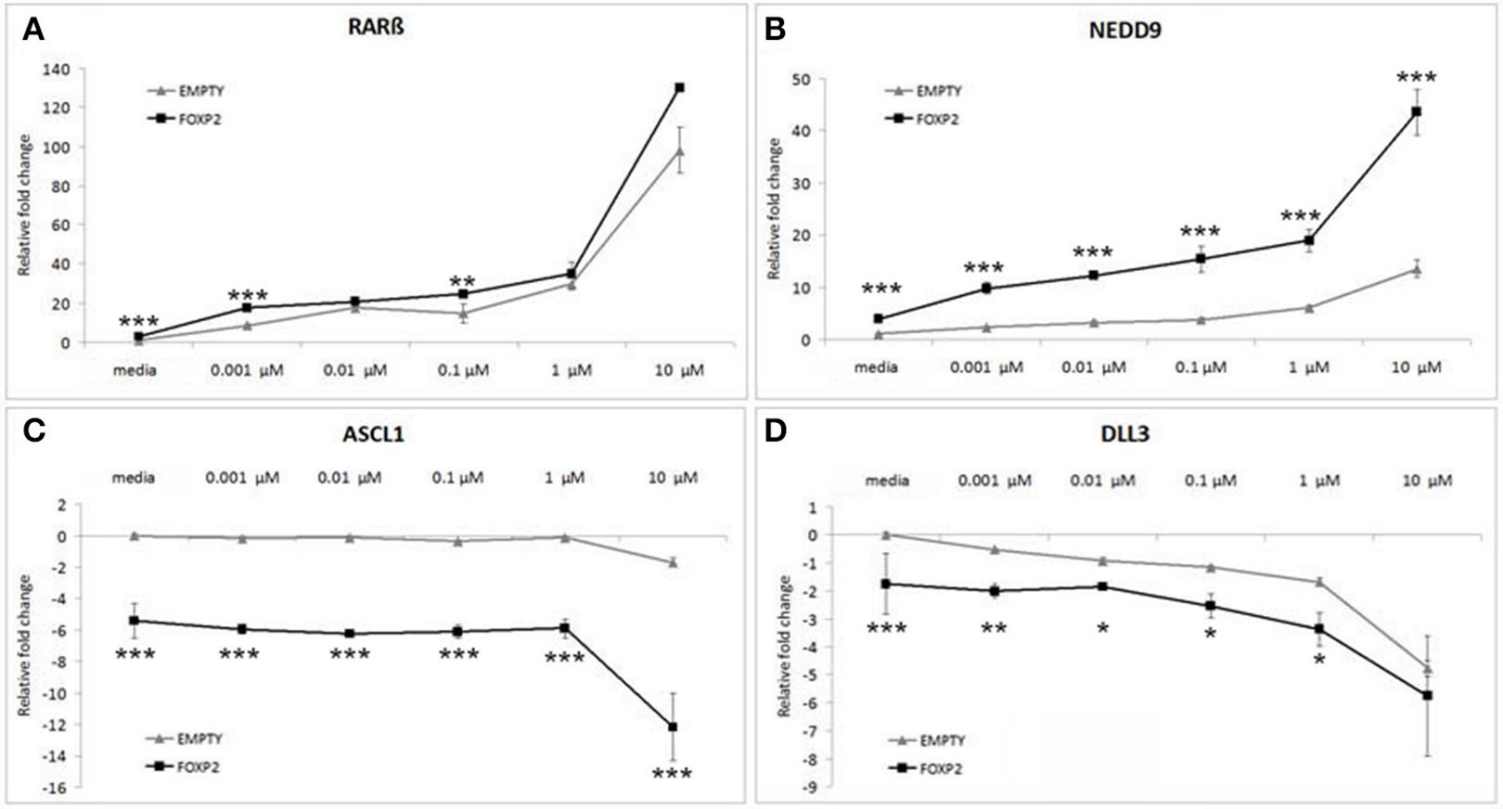
**FOXP2 expression makes cells more responsive to retinoic acid**. FOXP2 expression influences the cellular response to RA. Gene expression changes were measured in control (EMPTY) or FOXP2 expressing cells following treatment with varying levels of RA doses; ranging from 0.001 to 10 μM RA. Low serum media (“media”) containing 2% FCS acted as the baseline condition. In both EMPTY and FOXP2 cells, exposure to RA induces the expression of *RAR*β and *NEDD9* **(A,B)**, and represses *ASCL1* and *DLL3* **(C,D)**. The effect on gene expression levels of adding RA is greater in cells that are expressing FOXP2 positive cells, i.e., larger increases or decreases in expression are observed in the FOXP2 positive cells compared to the control cells. This effect is particularly striking for *ASCL1* and *NEDD9* at the highest RA concentration. Data are the average of three biological replicates expressed as mean ± standard deviation. Significant difference between all groups was calculated using an ANOVA test followed by *post-hoc* Bonferroni calculation and significance is given for the differences between the EMPTY and FOXP2 groups where ^*^p < 0.05, ^**^p < 0.01, ^***^p < 0.001.

Control (EMPTY) and FOXP2 expressing cells were treated with varying RA doses; low serum media alone (media) or low serum media containing a range of RA doses from low (0.001 μM) to high (10 μM) concentration. The lowest dosage had previously been shown to be sufficient to induce expression changes in SHSY5Y cells (Urban et al., 2010). Indeed, all doses of RA were able to significantly upregulate *RAR*β and *NEDD9* and to downregulate *DLL3* in the control (EMPTY) cell lines (Figure 4). Significant downregulation of *ASCL1* in control cells could only be observed after high dose (10 μM) RA treatment in control cells (Figure 4C). In the FOXP2 expressing cells, the molecular changes occurring in response to RA treatment were significantly stronger than in controls. Both *RAR*β and *NEDD9* were more strongly induced by a range of RA doses in FOXP2 expressing cells compared to control cells and this effect was particularly striking for NEDD9 at the highest dose (10 μM RA). FOXP2 strongly downregulated *ASCL1* in an RA independent fashion until cells were exposed to high dose RA (10 μM), at which point significantly stronger repression of *ASCL1* was observed by the combination of FOXP2 plus RA than either treatment alone. The combination of FOXP2 expression and RA treatment also enhanced the repression of *DLL3* at most RA doses.

Given that the effect of expressing FOXP2 in the presence of RA produced greater magnitude effects on gene expression levels than either treatment alone, we suggest that the presence of FOXP2 is making these cells more sensitive to retinoic acid levels, i.e., FOXP2 may alter the internal state of cells to allow a larger induction of signaling via downstream networks in a dose dependent manner.

### FOXP2 promotes increased neurite outgrowth in response to retinoic acid

**Figure 5:**
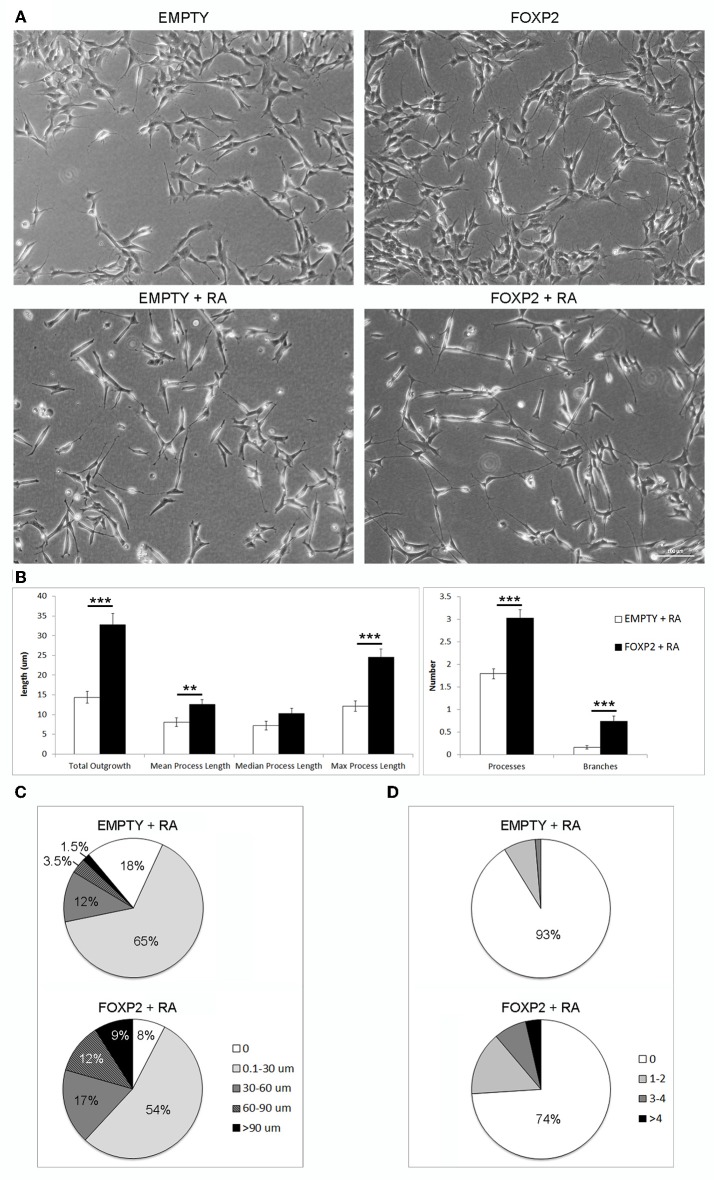
**FOXP2 promotes increased neurite outgrowth in response to retinoic acid. (A)** Example brightfield pictures demonstrating increased neurite growth in FOXP2 positive cells compared to EMPTY control cells both in normal growth media (upper panels) or after RA treatment for 3 days (lower panels) **(B)** SHSY5Y control (EMPTY) or FOXP2 cells were exposed to 10 μ M RA for 3 days before measurement of neurite length and complexity. FOXP2 expression resulted in highly significant increases in total outgrowths (*p* = 1.92E-08), mean process length (*p* = 7.36E-03) and max process length (*p* = 1.01E-06) as well as the number of cell processes (*p* = 3.11E-09) and branches (*p* = 1.76E-06). Data are the average of three biological replicates (*N* = 228 EMPTY cells and 223 FOXP2 cells) expressed as mean ± s.e.m. Statistical significance was assessed using students *t*-tests (two-tailed). ^**^p < 0.01, ^***^p < 0.00001. **(C)** Total outgrowths in EMPTY vs. FOXP2 cells. The majority (83%) of EMPTY (control) cells had either no measurable neurites, or “short” outgrowths (i.e., <30 μm total outgrowths). By comparison, this number was only 62% for FOXP2 expressing cells. FOXP2 expressing cells were far more likely to show long neurite growth, with 20% of cells having “long” neurites (totalling more than 60 μm), whereas only 5% of EMPTY cells had growth at or above this length. **(D)** FOXP2 significantly increases the number of secondary branches per cell. The majority of control cells (EMPTY) had no secondary branchpoints (93%), whereas when FOXP2 was expressed, only 74% of cells had no branches. 7% of EMPTY cells had between 1–4 branches, while 22.5% had 1–4 branches (compared to 9% in EMPTY cells) and 3.6% of FOXP2 expressing cells and more than four branches respectively. EMPTY cells never displayed more than four branches. Data are the average of three biological replicates (*N* = 228 EMPTY cells and 223 FOXP2 cells).

We have previously shown that endogenous expression of murine Foxp2 in neurons promotes the growth and branching of neurites in the mouse (Vernes et al., 2011). SHSY5Y FOXP2 cells also displayed more neurite growth than their control (EMPTY) counterparts when growing in normal growth media (Figure 5A, upper panels). Furthermore, retinoic acid differentiation of SHSY5Y cells induces the growth of long, extensively branched neurites (Figures 1A,B)(Voigt and Zintl, 2003; Lopes et al., 2010). Given the increased molecular response to retinoic acid exposure in FOXP2 positive cells, we investigated whether expression of FOXP2 affected the induction of neurite outgrowth by retinoic acid.

In order to assess the effect of FOXP2 on neurite outgrowth and branching, we exposed cells to retinoic acid to initiate neurite formation in SHSY5Y FOXP2 and control (EMPTY) cells. RA treated cells that expressed FOXP2 showed more neurite growth than the control cell line (EMPTY) (Figure 5A, lower panels). FOXP2 expression significantly increased the number and length of protrusions that grew in response to RA treatment (Figure 5B, left panel). The majority of control (EMPTY) cells (83%) had either no outgrowths or “short” outgrowths (<30 μm) following RA exposure (Figure 5C, upper panel). In contrast, only 62% of FOXP2 expressing cells were in the no outgrowth or “short” outgrowth category. FOXP2 expressing cells were more likely to have “long” outgrowths (i.e., protrusions exceeding 60 μm) (Figure 5C, lower panel).

FOXP2 expression significantly increased the number of branches observed per cell (Figure 5B, right panel). Processes in the majority of control (EMPTY) SHSY5Y cells were not branched and no EMPTY cells had more than four branches (Figure 5D, upper panel). FOXP2 expression increased branching, with a higher proportion of cells displaying branched outgrowths, a small number displaying as many as 14 branches (Figure 5D, lower panel). This illustrates that FOXP2 drives growth and branching of neurites during retinoic acid induced differentiation, and suggests that at both a molecular and a morphological level, FOXP2 expressing cells show increased responsiveness to retinoic acid.

### FOXP2 expression impairs neuronal cell migration

**Figure 6:**
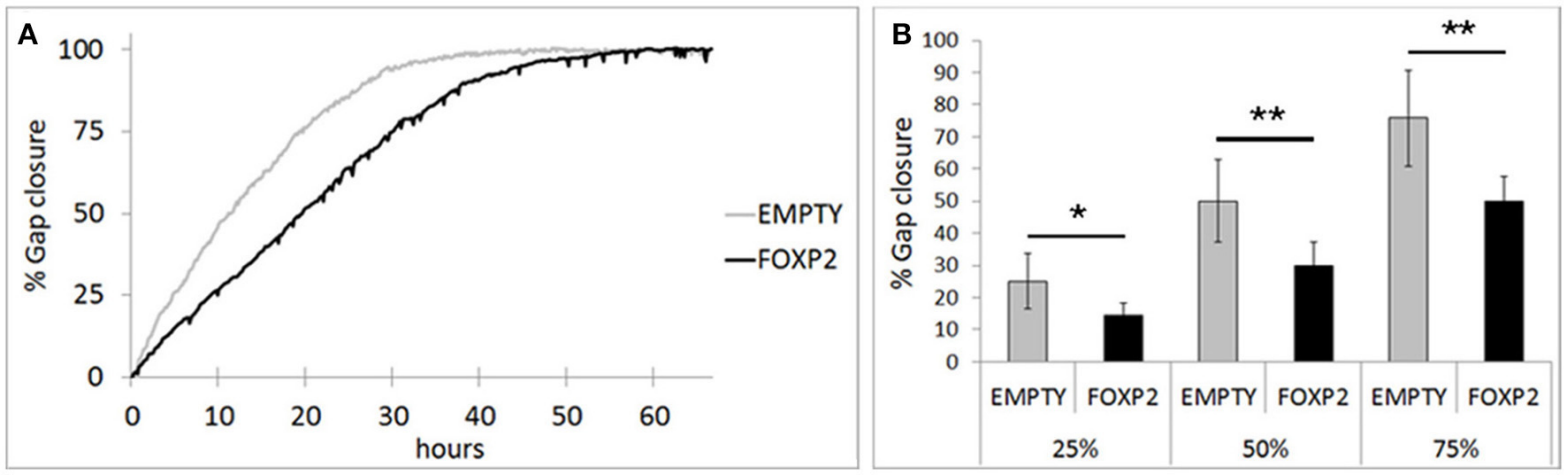
**FOXP2 affects the rate at which SHSY5Y cells can migrate**. Invasion assays were used to assess the migration of SHSY5Y cells expressing FOXP2 compared to control cells that are FOXP2 negative (EMPTY). Cells expressing FOXP2 migrate more slowly into the empty space. **(A)** Gap closure for EMPTY and FOXP2 cells over 72 h timecourse. Cells expressing FOXP2 did eventually close the gap, but did so at a later timepoint, demonstrating a quantifiably slower migration speed compared to control cells. **(B)** The average invasion frequency was calculated once EMPTY cells had reached 25, 50, and 75% gap closure. At each timepoint, FOXP2 expressing cells had completed significantly less gap closure (14, 30, and 50% closure respectively). Data is the average of 6 replicates expressed as mean ± standard deviation. Statistical significance was assessed using students *t*-tests (two-tailed). ^*^p < 0.05, ^**^p < 0.01.

RA induced differentiation reduces migration/invasion of SHSY5Y cells (Voigt and Zintl, 2003; Messi et al., 2008) and controlling the migration of cells in the brain is essential to establishing neuronal circuitry during development (Marin et al., 2010). Here, we assessed the effect of FOXP2 expression on SHSY5Y migration in gap closure assays. Using time lapse microscopy, we measured gap closure speed by EMPTY and FOXP2 cells. We found that FOXP2 expression reduced cell migration (Figure 6A). The effect of FOXP2 on migration was highly significant when assessing time to gap closure (Figure 6B). When control cells had reached 25, 50, and 75% gap closure, FOXP2 positive cells had reached only 14, 30, and 50% closure—a significantly reduced migration rate. Thus, in addition to promoting molecular and morphological features of differentiation, FOXP2 expression results in reduced speed of migration, a key feature of retinoic acid induced neuronal differentiation.

## Discussion

*FOXP2* has garnered significant attention due to its importance for normal speech and language development in humans. FOXP2 acts as a transcription factor and a diverse list of putative targets have been identified in a range of model systems, suggesting a role for FOXP2 in cellular processes such as neurite outgrowth (Vernes et al., 2011) and synapse development (Schulz et al., 2010; Sia et al., 2013). Indeed, mouse and songbird models of FOXP2 have revealed important roles in synaptic plasticity and neuronal connectivity (Haesler et al., 2007; Groszer et al., 2008; French et al., 2012; Murugan et al., 2013). However, it is still unclear how transcriptional programs controlled by FOXP2 affect neuronal development. In this study we demonstrate the importance of FOXP2 for human neuronal differentiation using a neuron-like model system.

We demonstrated that in normal growth media, FOXP2 increased the expression of neuronal markers such as *MAP2, DCX*, and *mir-9* and directed a transcriptional program that was consistent with retinoic acid induced differentiation. In addition to these molecular effects, we showed that FOXP2 induces phenotypic changes characteristic of neuronal differentiation such as increased neurite outgrowth and reduced migration. Neuronal migration and neurite outgrowth are tightly linked and many of the genetic mechanisms underlying these processes are shared (Marin et al., 2010). Throughout development, neurons migrate along tightly regulated paths to reach their correct destination before extending their processes to form connected neuronal circuitry. Control of both neuronal cell migration and neurite outgrowth are thus essential for normal brain development and defects in outgrowth or migration can lead to neurodevelopmental disorders such as intellectual disability and have been linked to disorders involving language impairment such as autism and dyslexia (Mcmanus and Golden, 2005; Paracchini et al., 2007; Wegiel et al., 2010; Bellon et al., 2011; Liu, 2011; Carrasco et al., 2012; Carrion-Castillo et al., 2013).

*In vivo* studies have illustrated the importance of FOXP2 for normal brain development. Individuals carrying heterozygous mutations of *FOXP2* display a complex speech and language disorder phenotype involving impaired articulation caused by an inability to coordinate the complex sequences of orofacial muscle movements required during speech (developmental orofacial dyspraxia; OMIM 602081), accompanied by both expressive and receptive linguistic and grammatical processing defects (Vargha-Khadem et al., 1995, 1998; Watkins et al., 2002a). Corresponding differences in brain activation in language related areas during linguistic tasks has been observed in these affected individuals, suggesting that disruption of *FOXP2* is causing subtle network connectivity and/or activation differences (Watkins et al., 1999, 2002b; Liégeois et al., 2003). However, it is still poorly understood how the molecular functions of FOXP2 can produce such a specific phenotype related to human speech and language circuitry. An intriguing finding of our study is that FOXP2 positive cells are more sensitive to retinoic acid treatment. Cells that both express FOXP2 and were RA treated displayed increased responses compared to either treatment alone. Our findings suggest that the transcriptional program enacted by FOXP2 alters the molecular composition of neurons, allowing these cells to respond differently to environmental retinoic acid cues driving neuronal differentiation. Retinoic acid signaling regulates proliferation, migration and differentiation of cells and is extremely important for normal brain development, contributing to forebrain, hindbrain and spinal cord patterning (Gudas, 1994; Rhinn and Dolle, 2012). RA induction of neuronal differentiation is mediated by a combination of morphogen signaling gradients acting on cells during a refractive “sensitive period” in which the appropriate gene expression changes can be induced to produce different neuronal fates (Sive et al., 1990; Holson et al., 2001; Maden, 2002; Yamamoto et al., 2003; Luo et al., 2004; White et al., 2007; Rhinn and Dolle, 2012). FOXP2 is only expressed in a subset of neurons in select brain regions, such as deep layer cortical neurons, Purkinje cells of the cerebellum and a subset of medium spiny neurons (MSNs) of the striatum (Ferland et al., 2003; Lai et al., 2003). The importance of having FOXP2 expression in these specific neuronal populations during development is not understood. However, given the findings presented here, FOXP2 may sensitize developing neurons to RA and thus program these subsets of neurons to respond differently to environmental RA cues than surrounding cells. In future, it will be of great interest to determine if this could specifically affect the differentiation and/or incorporation of FOXP2 positive cells into functioning neuronal circuitry, such as those subserving language.

The increased sensitivity to RA is likely mediated by FOXP2 inducing transcriptional changes across a range of molecules that we identified as acting downstream of both retinoic acid and FOXP2. This internal change of cellular components may collectively result in a greater response to retinoic acid levels. Mechanistically this may be occurring by affecting the classical RA pathway, or by affecting the non-genomic RA pathway (or potentially a combination of the two).

The classical RA pathway involves retinoic acid entering the nucleus and binding to transcription factors such as the retinoic acid receptors (RAR) (Gudas, 1994; Balmer and Blomhoff, 2002). Cellular retinoic acid binding protein II (CRABPII) transports intracellular retinoic acid into the nucleus so that it can interact with RARs (Gudas, 1994). Thus, CRABPII is able to modulate the cellular response to RA, the more CRABPII that is present, the more environmental RA can be transported into the nucleus (Delva et al., 1999; Dong et al., 1999). Once in the nucleus, RA binds to receptors such as RARβ to induce conformational changes that result in the differential regulation of downstream target genes and induce neuronal differentiation and increased neurite outgrowth (Gudas, 1994; Puttagunta et al., 2011; Al Tanoury et al., 2013; Rochette-Egly, 2014). Both CRABPII and RARβ have previously been shown to be strongly upregulated by exposure to retinoic acid (Hewson et al., 2002) and we observed the same effects in our system. Furthermore, we found that that both of these critical modulators of the classical RA pathway were upregulated by FOXP2 in normal growth media. Thus, we can see how FOXP2 causes an increase in the pool of both CRABPII and RARβ with which RA can interact. Conceivably this could lead to greater magnitude responses either at a gene expression or phenotype level being evoked by RA treatment in FOXP2 positive cells compared to FOXP2 negative (EMPTY) counterparts.

FOXP2 may also affect the non-genomic RA pathway which involves the rapid activation of kinase signaling pathways, such as MAPK or ERK as well as post-transcriptional control of gene expression (Rochette-Egly, 2014). These effects can again be mediated by RAR's, however the pool of RARs associated with the non-genomic pathway are not localized to the nucleus and thus do not directly mediate transcriptional changes (Al Tanoury et al., 2013; Rochette-Egly, 2014). Non-genomic effects of RA have been observed in hippocampal neurons where synaptically localized RARα interacts with mRNA to control translation levels in an RA dependent fashion, leading to altered synaptic plasticity (Aoto et al., 2008; Sarti et al., 2013). RARβ is not expressed in the hippocampus and thus it is not yet clear if it is able to perform similar synaptic functions to RARα in other tissues, however in primary striatal neurons of the developing mouse brain we have observed localization of the RARβ protein at synapses (data not shown), suggesting it has the potential to modulate synaptic protein levels in a similar fashion to RARα. By increasing the RARβ levels, FOXP2 may again be influencing the pool of molecules that can respond to RA exposure and thus the level of signaling through the non-genomic RA pathway.

Interestingly, *RAR*β has been shown to be essential for the normal patterning of the postnatal striatum (Liao et al., 2008), and both *RAR*β and *FOXP2* are highly expressed in the striatum, throughout development and into adulthood (Ferland et al., 2003; Lai et al., 2003; Takahashi et al., 2003; Liao et al., 2005). The striatum represents a site of pathology both structurally and in measures of functional activation during language related processing tasks in speech/language disorder patients carrying *FOXP2* mutations (Vargha-Khadem et al., 1998; Watkins et al., 2002b). Given the upregulation of *RAR*β by FOXP2, it will be of interest to determine if striatal patterning occurs in a FOXP2 dependent fashion and how patterning is affected by FOXP2 mutation. The importance of RA signaling for normal striatal development and the interplay between FOXP2 and RA responsive pathways, may indicate a neurogenetic mechanism by which FOXP2 is able to produce subtle but specific phenotypic effects in language related areas of the brain such as the striatum.

Retinoic acid signaling is also important for the specification of neuronal identity in the developing brain. In the neural tube, RA directs the differentiation of cells into serotonergic hindbrain or V3 spinal cord neurons and *ASCL1* (a proneural transcription factor also known as *MASH1*) has been shown to be the crucial downstream molecule directing this cell fate switch (Jacob et al., 2013). We found that *ASCL1* was strongly downregulated by either FOXP2 or by retinoic acid induced differentiation. Furthermore, *ASCL1* downregulation following RA treatment was significantly enhanced in FOXP2 expressing cells compared to controls. Another FOX family member, FOXO3, has been shown to repress *ASCL1* to drive the switch from neural progenitor cell to differentiated neuron (Webb et al., 2013). In these cells ASCL1 and FOXO3 have been shown to bind to enhancers of a large number of overlapping genes in a competitive manner (Webb et al., 2013). Given the strong downregulation of *ASCL1* by FOXP2 that we observed, coupled with the high conservation in the FOX family of both the FOX DNA binding domain and the core DNA motif recognized by these transcription factors, it is plausible that a similar model of action applies to FOXP2 positive neurons. Comparing our previously identified FOXP2 target genes (Spiteri et al., 2007; Vernes et al., 2007, 2011) with the ASCL1 targets from Webb et al. (2013), we found a very high degree of overlap (*N* = 387). These genes, putatively regulated by both FOXP2/ASCL1, displayed a significant over-representation of genes involved in neuronal differentiation (adjP = 2.78e-10) such as *WNT5A, EFNB2, SEMA3A/6A*, and *NRP2* (Vernes et al., 2007, 2011). Hence the regulation of *ASCL1* by FOXP2 may be a key step in driving differentiation in FOXP2 positive neurons.

We have shown that both molecular and phenotypic features of human neuronal differentiation can be promoted by the FOXP2 protein. In addition to upregulating classical neuronal markers, FOXP2 regulated the expression of a large number of differentiation responsive genes in a manner that was consistent with a more differentiated neuronal state. This transcriptional program is accompanied by phenotypic changes that occur during differentiation, i.e., increased neurite outgrowth and reduced neuronal migration. Intriguingly, our study suggests that FOXP2 causes cells to be more responsive to retinoic acid exposure. The mechanism for this sensitivity is likely related to FOXP2's ability to control the expression of retinoic acid responsive genes and may present a novel mechanism for controlling cell type specific differentiation patterns in the developing brain. In the future, it will be essential to determine *in vivo* if FOXP2 controls the responsiveness of neurons to RA during development. These findings demonstrate the importance of FOXP2 for human neuronal differentiation and illustrate how mutations could lead to aberrant differentiation of neurons in the developing brain. This link between FOXP2 function and RA pathways may point to a novel role for retinoic acid signaling in the development of language related neuronal circuitry.

### Conflict of interest statement

The authors declare that the research was conducted in the absence of any commercial or financial relationships that could be construed as a potential conflict of interest.
